# Kentucky State Dental Society

**Published:** 1888-08

**Authors:** 


### Kentucky State Dental Society.

The eighteenth annual meet of this society was called to order at two o'clock, P. M., June 5th, in the Louisville Dental College, Louisville; President W. S. Smith in the chair. The President delivered an address reviewing the progress made by the profession during the past twenty years.

*Dr. Hooper*—Moved that the courtesies of the floor be extended to the physicians of the city. The motion was adopted. The hours of sessions were fixed at from 9 to 12 A. M., 2 to 5 P. M., and the evening session from 8 o'clock until adjournment. The President announced that a gentleman was present who possessed an anæesthetic, the properties of which he wished to explain with a purpose of selling the secret. The gentleman was not allowed to exhibit his nostrum.

Excellent essays were read on "Inflammation" by Dr. Henry D. Eggers, of Louisville, and on "Antiseptics in the Oral Cavity" by Dr. L. L. King, of Henderson. The resignations of Dr. A. Wilkes Smith, of Richmond, and Dr. A. O. Rawls, of Lexing- from the State Board of Examination were offered and accepted.

*Dr. F. M. Harris*, New Albany, Ind., read a paper on "The Use of Cement in Molars."

The following charges of unprofessional conduct were made against Dr. J. Hooper, of Louisville:

"*To the Board of Censors of the Kentucky State Dental Association:* GENTLEMEN—We, members in good standing of the Kentucky State Dental Association, prefer charges against J. Hooper, of the city of Louisville, a member of this Association, for unprofessional conduct and violation of the Code of Ethics, and request your Board to examine into these charges. Said charges consist in the violation of Sections 1 and 3 of Article 2, of the Code of Ethics, to-wit:

"*First*—That on a certain day Dr. Hooper did, in his office, state to a patient in his chair, and to another awaiting, that he should have to remove a pulp from a tooth and put in another pulp; whereupon, being questioned by the lady in waiting as to the possibility of such an operation, said Hooper presented a box of gutta percha root fillings, well known and used by the profession, stating that they were the nerves to be used, and that he is the only dentist in Louisville who could perform said operation. All of which was calculated to mislead, and is false in fact.

"*Second*—Furthermore, that at the meeting of the American Dental Association, held at Niagara Falls, N. Y., in 1886, said Hooper did read a paper entitled 'Case No. 2,' coming under the head of surgical cases, in which he reflected upon the professional skill and ability of a member of this Association in good standing.

"*Third*—Furthermore, that said Hooper did, upon divers occasions, state to Prof. A. Wilkes Smith, of the Louisville College of Dentistry, that he was the only operator in Louisville who could perform certain operations well known to the profession, and did exhibit well-known instruments and apparatus, stating that he was the inventor of certain improvements thereto.

"*Fourth*—Furthermore, that said Hooper did present a pneumatic plugger to Dr. Junius Alexander, and stated that he was the inventor thereof, the patterns of which were obtained from a plugger in the possession of Dr. Tileston.

"*Fifth*—Furthermore, that said Hooper did, in a case in which Dr. Tileston had extracted a tooth for purposes of regulation, and which afterwards fell into the hands of Dr. Hooper, attempt to persuade said patient that Dr. Tileston was guilty of malpractice, and had tried to persuade the patient to bring suit against Dr. Tileston for malpractice.

A. WILKES SMITH,
F. PEABODY,
HENRY D. EGGERS.

"*Sixth*—In addition, I state that Dr. Hooper made the statement to Dr. F. C. Greene, of New Albany, Ind., that he was a graduate of the Ohio Dental College.

F. PEABODY.

The Board of Censors, under the order of the President, took charge of the paper and at once retired to another room for the purpose of investigating the charges, and nothing more was heard from it, except an occasional call for a witness, during the afternoon session.

*Dr. H. B. Tileston*—Was next in order on "Plastic Filling,"

but failed to respond when called for, consequently one of the voluntaries was named—Dr. W. P. McQuown, of Georgetown—whose subject was "The Duty of a Dental Association." Dr. McQuown, however, announced that he had changed his mind, and would read a short paper on "The Treatment of Pulpless Teeth," which he did, eliciting commendations. His document was very brief and to the point. It was discussed by Drs. Tileston, Edwards, Alexander, Harlan, of Chicago; Rosser, Rawls, and others. This discussion was very interesting, and was carried on almost to the hour of adjournment.

The Association adjourned at 5 o'clock until the evening hour.

EVENING SESSION.

The night session began at 8.30 o'clock. Dr. Rawls suggested that, as Dr. Taft, a member of the association, had suffered misfortune in the death of his wife, it would be proper for the body to express its sympathy. He moved the appointment of a committee for that purpose, which was unanimously carried, and Drs. Rawls, Edwards and Peabody were appointed.

Dr. Edwards, in view of the meeting of the Southern Dental Association and the American Dental Association on August 28, invited all members of the State Association to attend the joint meeting of those two bodies.

A motion was made that the chair appoint a Committee on Reception, select a speaker to deliver an address of welcome to the two associations named, and also name a speaker to deliver an address in behalf of the Kentucky State Dental Association. After considerable discussion the motion was adopted, and the chair designated Dr. Rawls as the speaker in behalf of the State Association.

Drs. Edwards, of Louisville, Smith, of Richmond, Rosser, of Elizabethtown, and King of Henderson, were appointed a committee to arrange sub-committees, whose business it shall be to make the necessary arrangements for the reception of delegates to the joint meeting in August. The entire organization was constituted a Reception Committee.

Dr. Harris' paper on "The use of Cement in Molars" was brought up and discussed by several gentlemen, and proved quite interesting. At this juncture the Board of Censors appeared and submitted its report on the charges heretofore preferred against Dr. J. Hooper for violation of the Code of Ethics. The report was read as follows:

"We, your Committee, after due consideration and examination of all the proof attainable, in the case of Dr. J. Hooper, against whom charges have been preferred by Drs. A. Wilkes Smith, F. Peabody and Henry D. Eggers, beg leave to report that we sustain the following charges—Nos. 1, 3, 5 and 6.

J. H. BALDWIN,
WM. VAN ANTWERP,
L. A. KING.

Louisville, Ky., June 7, 1888."

The report of the board was, on motion, received, and a vote ordered on the penalty to be inflicted—the vote being on the highest penalty first. Drs. Reese and Baxter were appointed tellers.

The Board of Censors asked to be excused from voting, having sat as a jury in the matter, and, on motion duly made, recorded and carried, the members of that board were excused.

The tellers had but little work to do. Only those members were allowed to vote who had paid their dues, which, with the excusing of the board, cut the number cast down to fifteen. Thirteen of these were for expulsion and two against it. When the vote was announced, the President declared that Dr. Hooper was no longer a member of the association, which announcement was received with applause.

*Dr. W. E. Baxter*—Read an entertaining paper on "Temperaments," which was followed by an interesting address by Dr. W. S. Smith, of New Castle, on "Danger Arising from the Treatment of Tuberculous Patients." Dr. Smith declared that medical investigation had proved conclusively that consumption is contagious by inhalation and continued contact with persons suffering from the disease. He said that dentists are frequently called upon to operate on the teeth of consumptives, and that the utmost caution should be used lest the operator be infected with the terrible malady. He recommended that the best disinfectants be used, and that the instruments be thoroughly cleansed and disinfected after use. Dr. Eggers denied that the disease was contagious, and declared that no proof could be adduced to show that it was. He declared that it is hereditary and can not be controlled. He thought the disease was syphilitic in its origin. Drs. Barns and Peabody declared that the disease is both hereditary and contagious, and thought that while dentists, from the fact that while at work they are constantly surrounded by disinfectants are less liable than other persons to contract the disease, still they should use the greatest precaution.

Last day one session only was held. A clinic on bridgework was held by Drs. Edwards and Baxter. Drs. G. S. Seymour and E. M. Ketting made application for membership in the association and were elected. The committee appointed to draft resolutions of sympathy for Dr. J. Taft offered its report, which was unanimously adopted.

The election of officers, committeemen and delegates was proceeded with, resulting as follows:

President—Dr. J. H. Baldwin, Louisville.

Vice-President—Dr. H. B. Tileston, Louisville.

Secretary—Dr. C. E. Dunn, Louisville.

Treasurer--Dr. J. F. Canine, Louisville.

Board of Censors--Dr. L. A. King, Henderson; Dr. W. E. Baxter, Frankfort.

Executive Committee--Dr. W. P. McQuown, Georgetown.

State Board of Examiners--Dr. C. V. Rosser, Elizabethtown; Dr. Van Antwerp, Mt. Sterling.

Delegates to American Dental Association--Drs. J. B. Alexander, L. A. King, C. V. Rosser, A. W. Smith, F. Peabody, J. F. Canine. Alternates--Drs. G. A. Foster, H. B. Tileston, E. M. Kettig, W. P. McQuown, W. Van Antwerp.

A vote of thanks was tendered the newspapers of the city and the Faculty of the Louisville Dental college for courtesies and favors. After this there was some little routine business transacted and the association adjourned to meet in Louisville, on the first Tuesday in June, 1889.

